# Effects of Antisolvent Treatment on Copper(I) Thiocyanate Hole Transport Layer in n-i-p Perovskite Solar Cells

**DOI:** 10.3390/molecules29184440

**Published:** 2024-09-19

**Authors:** Sehyun Jung, Seungsun Choi, Woojin Shin, Hyesung Oh, Nahyun Kim, Sunghun Kim, Namkook Kim, Kyuhyun Kim, Hyunbok Lee

**Affiliations:** Department of Physics, Kangwon National University, 1 Gangwondaehak-gil, Chuncheon-si 24341, Republic of Korea; nghf97@naver.com (S.J.); tmdtns619@naver.com (S.C.); swj452@gmail.com (W.S.); dkgncl@gmail.com (H.O.); qmfforghf455@naver.com (N.K.); tksxk1999@kangwon.ac.kr (S.K.); skarnr1109@naver.com (N.K.); qrqrafaf12@gmail.com (K.K.)

**Keywords:** perovskite solar cell, hole transport layer, CuSCN, antisolvent

## Abstract

Copper(I) thiocyanate (CuSCN) is considered an efficient HTL of low cost and with high stability in perovskite solar cells (PSCs). However, the diethyl sulfide solvent used for CuSCN preparation is known to cause damage to the underlying perovskite layer in n-i-p PSCs. Antisolvent treatment of CuSCN during spin-coating can effectively minimize interfacial interactions. However, the effects of antisolvent treatment are not sufficiently understood. In this study, the effects of five different antisolvents were investigated. Scanning electron microscopy and X-ray diffraction analyses showed that the antisolvent treatment improved the crystallinity of the CuSCN layer on the perovskite layer and reduced damage to the perovskite layer. However, X-ray and ultraviolet photoelectron spectroscopy analyses showed that antisolvent treatment did not affect the chemical bonds or electronic structures of CuSCN. As a result, the power conversion efficiency of the PSCs was increased from 14.72% for untreated CuSCN to 15.86% for ethyl-acetate-treated CuSCN.

## 1. Introduction

Perovskite solar cells (PSCs) have attracted significant attention due to their low fabrication costs, excellent light absorption ability, and tunable band gap [1,2,3,4,5,6]. The power conversion efficiency (PCE) of PSCs has increased remarkably, reaching up to 26% [7]. Generally, PSCs are fabricated with a multilayer structure, and charge transport layers are interposed between the perovskite layer and electrode to facilitate carrier transport. 2,2’,7,7’-tetrakis[N,N-di(4-methoxyphenyl)amino]-9,9’-spirobifluorene (spiro-OMeTAD) is the most popular hole transport layer (HTL) material [8,9]. However, its high cost poses a challenge to its commercialization. In addition, spiro-OMeTAD must be used with bis(trifluoromethane)sulfonimide lithium salt and 4-tert-butylpyridine additives for p-doping, which complicates device operation and causes long-term instability. An alternative approach is the use of inorganic HTLs [10,11,12,13]. Among the various inorganic HTL materials, copper(I) thiocyanate (CuSCN) is an efficient option due to its affordability, abundance, high hole mobility, thermal stability, and high work function, and it has been used in various optoelectronic devices [14,15,16,17,18,19,20,21,22,23]. Therefore, extensive research has been conducted on the development of PSCs using the CuSCN HTL.

The CuSCN layer is generally deposited by a solution process, which is advantageous from the perspective of low-cost device fabrication, although vacuum-evaporated CuSCN exhibits excellent hole transport ability [24]. However, due to its relatively low solubility, CuSCN must be dissolved in strong solvents, such as diethyl sulfide (DES), dipropyl sulfide, ammonium hydroxide, and dimethyl sulfoxide (DMSO), posing difficulties in various applications. In particular, these solvents can partially dissolve the underlying perovskite layer in n-i-p PSCs during CuSCN deposition, thereby degrading the optoelectronic properties of the devices [18,25,26,27,28].

To overcome this problem, antisolvent treatments of CuSCN have recently been investigated [29,30,31]. Antisolvents readily mix with the solvent but do not dissolve the solute. Therefore, antisolvent treatment during spin-coating facilitates rapid solvent removal and controls the crystal growth kinetics, thereby reducing the interaction with the underlying layer and improving the film crystallinity. In addition, the amount of trapped DES solvent residue, which is detrimental to hole transport, is effectively reduced. As a result, antisolvent-treated CuSCN films exhibited improved device performance. Such antisolvent treatments are also commonly used for the deposition of perovskite layers, which significantly enhance the light-absorbing and charge-transporting abilities [32]. However, despite many efforts towards CuSCN applications, a fundamental understanding of the material properties of antisolvent-treated CuSCN films is still lacking. For example, changes in the valence electronic structure of CuSCN films by antisolvent treatment, which significantly affect the energy level alignment and corresponding hole transport ability of devices [33,34], have not yet been investigated. Therefore, further studies are required to fine-tune the functionality of CuSCN.

**Figure 1:**
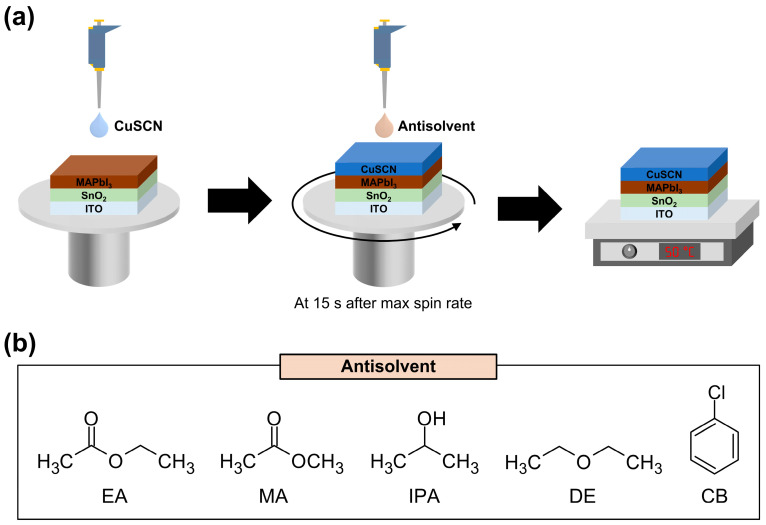
(**a**) Schematic of the CuSCN film deposition and antisolvent treatment processes and (**b**) chemical structures of the antisolvents.

In this study, we applied five different antisolvents [ethyl acetate (EA), methyl acetate (MA), 2-propanol (IPA), diethyl ether (DE), and chlorobenzene (CB)] during CuSCN deposition and investigated the changes in the film properties. A schematic of the CuSCN film deposition and antisolvent treatment processes is shown in Figure 1a. The chemical structures of the antisolvents are shown in Figure 1b. These solvents do not dissolve the underlying perovskite layer and are therefore suitable for use as antisolvents. The morphological characteristics, crystal structure, and electronic structure of the antisolvent-treated CuSCN films were extensively investigated using scanning electron microscopy (SEM), X-ray diffraction (XRD), and X-ray/ultraviolet photoelectron spectroscopy (XPS/UPS), respectively. Finally, PSC devices were fabricated using the antisolvent-treated CuSCN HTL, and the corresponding enhanced device performance was demonstrated.

## 2. Results and Discussion

**Figure 2:**
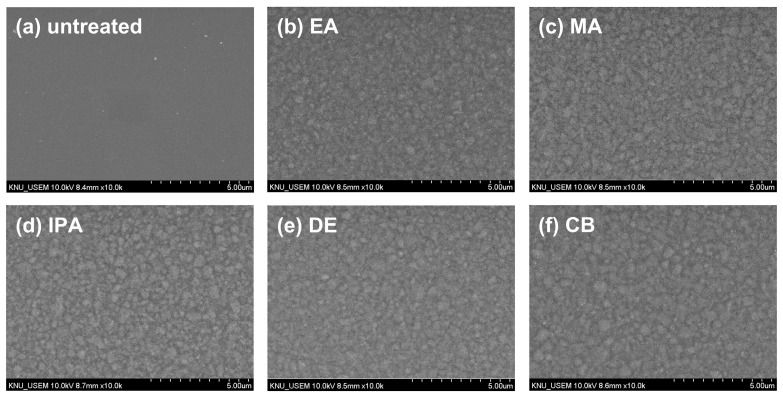
Top-view SEM images of CuSCN samples (**a**) without antisolvent treatment and with (**b**) EA, (**c**) MA, (**d**) IPA, (**e**) DE, and (**f**) CB antisolvent treatment prepared on ITO/SnO_2_/MAPbI_3_.

To investigate the changes in the morphology of the CuSCN film upon antisolvent treatment, top-view SEM images were captured. For the SEM and XRD measurements, CuSCN films were prepared on indium tin oxide (ITO)/SnO_2_/methylammonium lead triiodide (MAPbI_3_) to reflect the n-i-p PSC structure. Figure 2 shows the surface morphology of (a) untreated and (b) EA-, (b) MA-, (c) IPA-, (d) DE-, and (e) CB-treated CuSCN films. As shown in Figure 2a, the untreated CuSCN film had a smooth surface with very small grains. However, as shown in Figure 2b–f, the antisolvent-treated CuSCN films exhibited a significantly different morphology with larger grains. However, no significant differences were observed among the antisolvent-treated CuSCN films, indicating that the effects of the antisolvents were similar.

**Figure 3:**
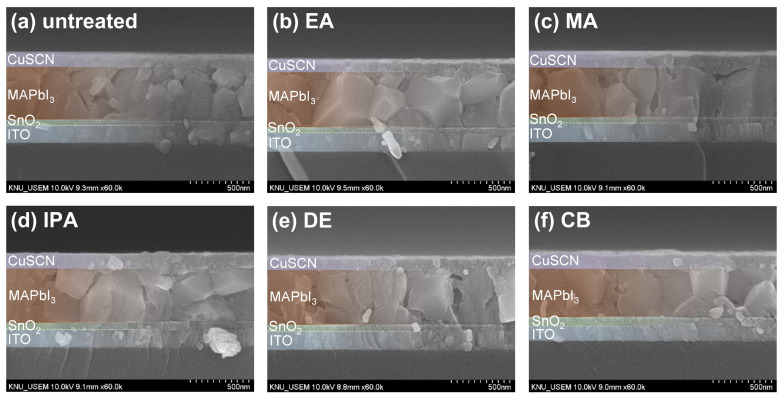
Cross-sectional SEM images of CuSCN samples (**a**) without antisolvent treatment and with (**b**) EA, (**c**) MA, (**d**) IPA, (**e**) DE, and (**f**) CB antisolvent treatment prepared on ITO/SnO_2_/MAPbI_3_.

Figure 3a–f show cross-sectional SEM images of the untreated and antisolvent-treated CuSCN films. The CuSCN, MAPbI_3_, SnO_2_, and ITO layers had distinctly different structures, and the interfaces are marked by dashed lines. It was expected that DES would cause damage to the perovskite layer during CuSCN deposition. However, no significant degradation was observed at the interface between the MAPbI_3_ and CuSCN layers upon visual inspection of the cross-sectional SEM images. In addition, no significant difference was observed at the MAPbI_3_/CuSCN interfaces between the untreated and antisolvent-treated samples. Furthermore, all samples were uniformly deposited without pinholes. These results are somewhat different from those previously reported [29], which clearly showed the formation of pinholes when CuSCN was deposited on the perovskite layer. A possible explanation is that the CuSCN had better wetting properties under our experimental conditions. However, it is important to note that this observation is based on visual inspection only and does not rule out the possibility of physical or chemical changes at the MAPbI_3_/CuSCN interface. The effect of DES on the perovskite layer is discussed in more detail later in the context of XRD. Meanwhile, we measured the thickness of each layer by analyzing the cross-sectional SEM images. Consequently, the thicknesses of the SnO_2_, MAPbI_3_, and CuSCN layers were approximately 50, 440, and 100 nm, respectively.

**Figure 4:**
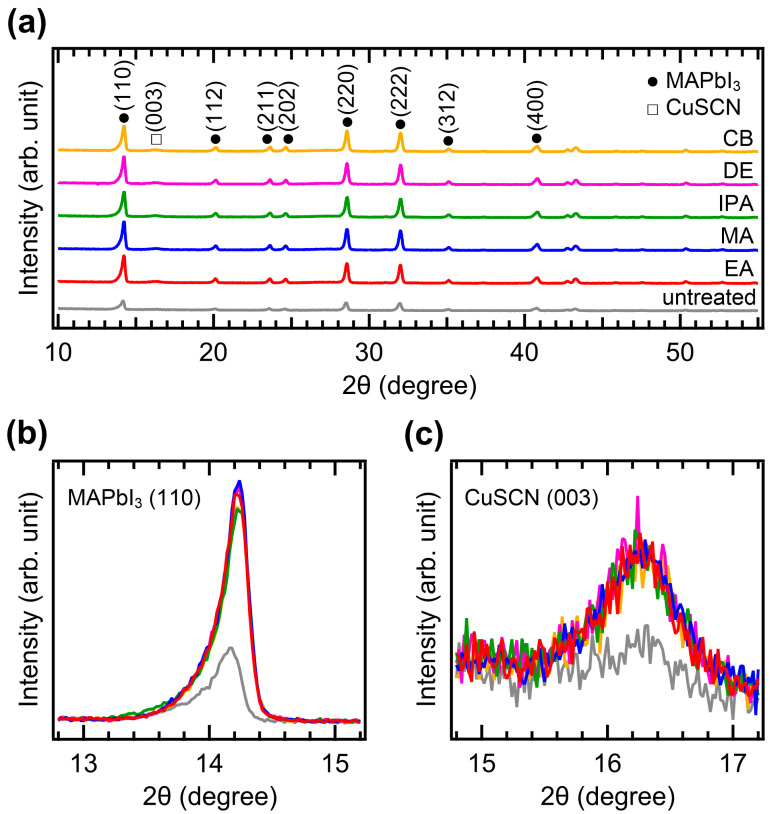
XRD patterns of the (**a**) wide, (**b**) MAPbI_3_ (110), and (**c**) CuSCN (003) regions of ITO/SnO_2_/MAPbI_3_/CuSCN films with different antisolvent treatments of CuSCN.

The crystallinity of CuSCN was analyzed by XRD measurements. Figure 4a shows the XRD patterns of the CuSCN films with and without antisolvent treatment. In all the XRD profiles, the characteristic peaks of MAPbI_3_ perovskite were observed at 14.2, 20.1, 23.6, 24.6, 28.6, 32.0, 35.1, and 40.8°, corresponding to the (110), (112), (211), (202), (220), (222), (312), and (400) planes, respectively [35]. However, the peak intensities of the antisolvent-treated CuSCN were higher than those of the untreated CuSCN. In addition, a CuSCN peak at 16.3° derived from the (003) plane was observed, which is in good agreement with a previous report (JCPDS No. 29-0581) [36]. To compare the XRD peak intensities in detail, the perovskite (110) and CuSCN (003) peaks were magnified, as shown in Figure 4b,c, respectively. Remarkable changes in the peak intensity due to antisolvent treatment were observed. In Figure 4b, the perovskite (110) peaks of all antisolvent-treated samples show significantly higher intensities than that of the untreated sample. The increased intensity of the perovskite peaks can be interpreted as a reduction in the damage caused to the perovskite layer by antisolvent treatment. This indicates that the DES solvent used for CuSCN deposition does indeed degrade the crystallinity of the underlying perovskite layer, although the damaged interface is not detectable in the cross-sectional SEM images (Figure 3). The improvement in perovskite crystallinity was a result of the rapid removal of the DES solvent by antisolvent treatment. As shown in Figure 4c, the CuSCN (003) peaks were also significantly higher in the antisolvent-treated samples than in the untreated sample. The higher intensity observed in the antisolvent-treated samples compared to that in the untreated sample was due to the improved crystallinity of CuSCN. This suggests that minimally damaged MAPbI_3_ surfaces may promote enhanced crystal growth of the subsequently deposited CuSCN. The presence of distinct CuSCN grains in the antisolvent-treated samples, as observed in the top-view SEM images (Figure 2), agreed well with the higher intensity of the CuSCN peaks observed in the XRD results. It was reported that the polarity of the antisolvent can influence the film properties [37]. In our study, the relative solvent polarities of EA, MA, IPA, DE, and CB were 0.228, 0.253, 0.546, 0.117, and 0.188, respectively (with water as a reference at 1.00) [38]. However, the morphology and crystallinity of the CuSCN films did not show significant differences. Thus, the polarity of the antisolvents did not have a major effect on the CuSCN film formed on MAPbI_3_.

**Figure 5:**
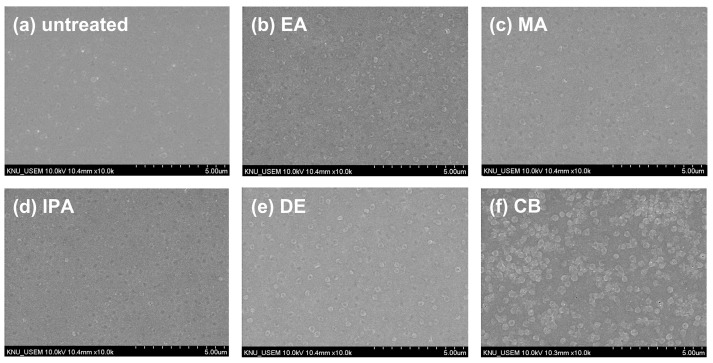
Top-view SEM images of CuSCN samples (**a**) without antisolvent treatment and with (**b**) EA, (**c**) MA, (**d**) IPA, (**e**) DE, and (**f**) CB antisolvent treatment prepared on ITO.

The properties of the CuSCN films were further investigated without the presence of the MAPbI_3_ layer. Although in n-i-p PSCs, the CuSCN HTL is deposited on the MAPbI_3_ layer, these analyses help to clarify the effects of antisolvent treatments on CuSCN film formation. Figure 5 shows top-view SEM images of ITO/CuSCN, with ITO as the reference substrate. In Figure 5a, the untreated CuSCN film shows a smooth surface with small grains. In contrast, Figure 5b–f show antisolvent-treated CuSCN films with relatively larger grains. The morphologies vary depending on the antisolvent used, with CB-treated CuSCN showing significantly larger grains. This behavior was different from the results observed for CuSCN on MAPbI_3_, shown in Figure 2. Therefore, the similar morphologies of antisolvent-treated CuSCN films on MAPbI_3_ could be attributed to interactions with the underlying MAPbI_3_ layer rather than the antisolvent itself.

**Figure 6:**
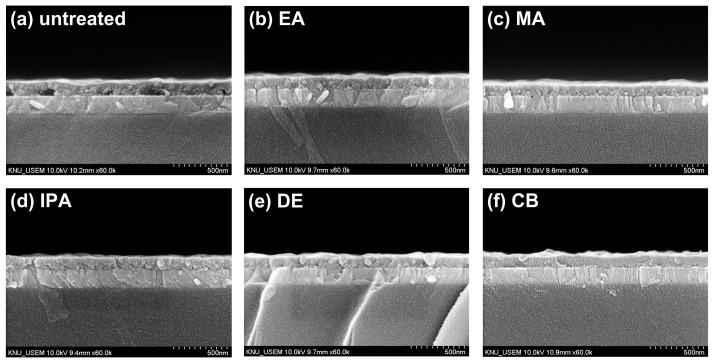
Cross-sectional SEM images of CuSCN samples (**a**) without antisolvent treatment and with (**b**) EA, (**c**) MA, (**d**) IPA, (**e**) DE, and (**f**) CB antisolvent treatment prepared on ITO.

Cross-sectional SEM images of ITO/CuSCN are shown in Figure 6. In Figure 6a, the untreated CuSCN film clearly contains pinholes at the ITO interface, while in Figure 6b–f, the antisolvent-treated CuSCN films show no noticeable pinholes. This was in contrast to the results observed for the MAPbI_3_ layer, where no clear distinction was found between untreated and antisolvent-treated CuSCN films. This also suggests that the interaction between CuSCN and MAPbI_3_ plays a critical role in CuSCN film formation. The thickness of the untreated CuSCN film was 124 ± 10 nm, while the antisolvent-treated CuSCN film measured 109 ± 6 nm, indicating that the antisolvent treatment slightly reduced the thickness of the CuSCN film.

**Figure 7:**
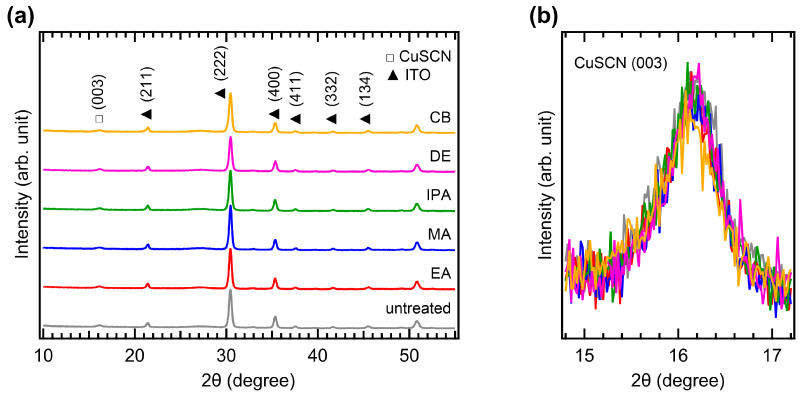
XRD patterns of the (**a**) wide and (**b**) (003) regions of ITO/CuSCN films with different antisolvent treatments of CuSCN.

The XRD patterns of CuSCN on ITO are compared in Figure 7, where Figure 7a,b show the full and CuSCN (003) regions of the ITO/CuSCN films, respectively. Unlike the XRD patterns of CuSCN on MAPbI_3_, the intensities of the CuSCN (003) peak remained unchanged regardless of the antisolvent used. The SEM and XRD results suggest that while the antisolvent treatment improves the uniformity of the CuSCN film by eliminating pinholes, it does not improve its crystallinity. The improved crystallinity of CuSCN on MAPbI_3_ may be due to changes in the interfacial interaction between the two materials. During CuSCN spin-coating on MAPbI_3_, DES can dissolve the MAPbI_3_ surface, forming a mixed layer of CuSCN and MAPbI_3_, which negatively affects the crystallinity of both. However, antisolvent treatment significantly reduces the interaction time between CuSCN and MAPbI_3_, allowing the mixed layer to form briefly, which may help to separate the CuSCN and MAPbI_3_ layers, resulting in improved crystallinity for both. Further detailed studies are needed to fully understand the interface formation between CuSCN and MAPbI_3_.

**Figure 8:**
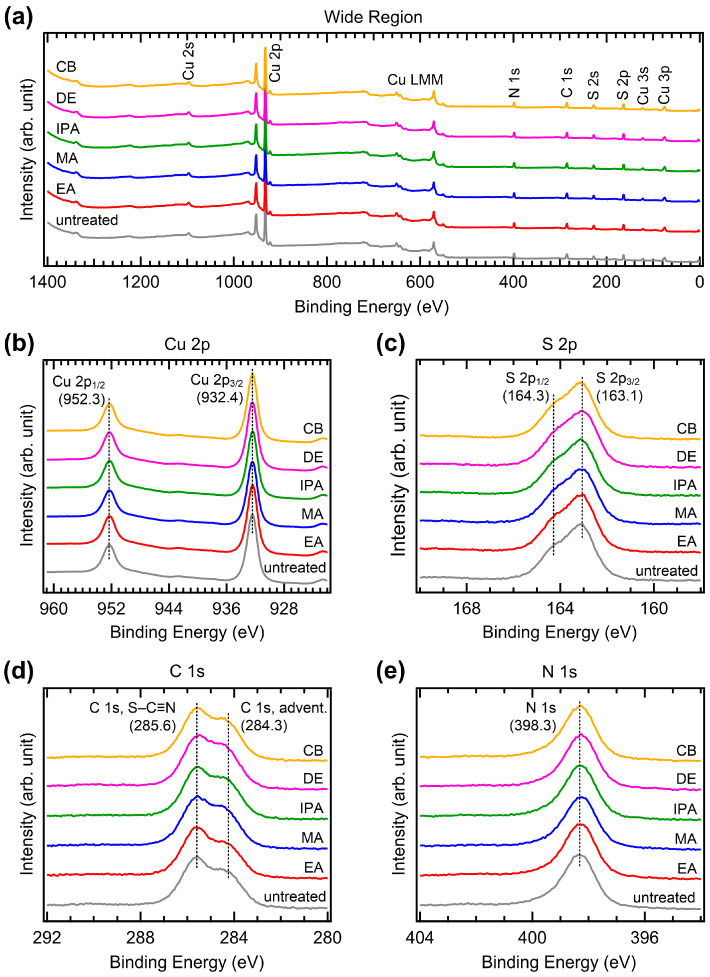
XPS spectra of the (**a**) wide, (**b**) Cu 2p, (**c**) S 2p, (**d**) C 1s and (**e**) N 1s regions of CuSCN films with and without antisolvent treatment.

To investigate possible changes in the chemical bonds induced by antisolvent treatment, we performed XPS measurements. Figure 8 shows the XPS spectra of the CuSCN films with and without antisolvent treatment. We first performed XPS measurements on CuSCN films on the ITO/SnO_2_/MAPbI_3_, which corresponded to the device structure. However, strong charging effects were observed due to the low conductivity of the substrate. Therefore, for the XPS (and UPS) measurements, the CuSCN films were prepared on an Au substrate. Figure 8a shows the wide-region XPS spectra. Cu, S, C, N, and Auger signals derived from CuSCN were observed; however, the Au signal was not observed due to the high thickness of CuSCN. In addition, no antisolvent-related atomic peaks (e.g., Cl in CB) were detected, indicating that there was no residual antisolvent. Almost identical spectral features were observed in all CuSCN samples. To check the spectral changes in detail, XPS spectra of the narrow regions for Cu 2p, S 2p, C 1s, and N 1s were obtained. As shown in Figure 8b, two Cu 2p_3/2_ and 2p_1/2_ peaks were observed at 932.4 and 952.3 eV, respectively, due to spin-orbit splitting. Both Cu 2p_3/2_ and 2p_1/2_ peaks were observed at the same binding energies, irrespective of antisolvent treatment. The peak intensities were also almost identical. As shown in Figure 8c, the S 2p_3/2_ and S 2p_1/2_ peaks were observed at 163.1 and 164.3 eV, respectively. The S 2p_3/2_ and 2p_1/2_ peaks were also observed at the same binding energies, with similar intensities in all samples. Similar results were observed in the C 1s and N 1s XPS spectra. As shown in Figure 8d, two C 1s peaks were observed to originate from the S–C≡N bond at 285.6 eV and adventitious C at 284.3 eV, respectively [39]. As shown in Figure 8e, the N 1s peak was observed at 398.3 eV. These peaks were observed to have the same characteristics in all samples. Therefore, it can be concluded that antisolvent treatment does not alter the chemical bonds in CuSCN. Notably, the choice of solvent significantly affects the chemical bonds [40]. However, antisolvent treatment only affects the morphology and crystallinity of the film and does not alter the chemical bonds.

**Figure 9:**
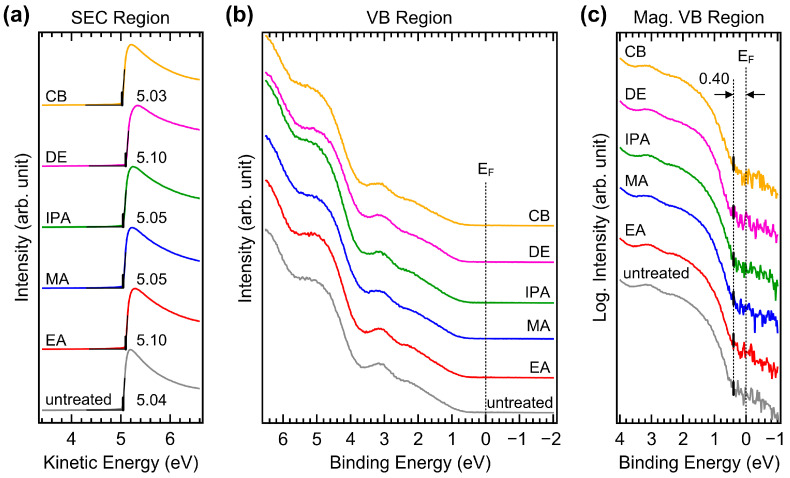
UPS spectra of (**a**) SEC and (**b**) VB regions of CuSCN films with and without antisolvent treatment. (**c**) UPS spectra of magnified VB region plotted on a log intensity scale of CuSCN films with and without antisolvent treatment. EF denotes the Fermi level.

UPS measurements were performed to investigate possible changes in the valence electronic structure. Figure 9a,b show the UPS spectra of the secondary electron cutoff (SEC) and valence band (VB) regions of CuSCN films with and without antisolvent treatment, respectively. A sample bias of −5 V was applied to record the SEC. The spectra of the SEC region were normalized and plotted against the kinetic energy scale such that the SEC position indicated the work function. The work function of the untreated CuSCN film was 5.04 eV. This high work function was beneficial for efficient hole transport to the anode. For the EA, MA, IPA, DE, and CB antisolvent treatments, the work functions ranged from 5.03 to 5.10 eV and were not significantly different. Several measurements were performed, and it was concluded that the small deviation was within the experimental error and not due to the choice of antisolvent. In the VB region, the spectral shapes were almost the same. For accurate determination of the VB maximum (VBM), the He I_β_ emission features were effectively removed from the measured spectra [41]. Because the VB of CuSCN has band-tail states, the VBM needed to be determined in terms of log intensity. Figure 9c shows magnified spectra of the VB region on a log intensity scale. The VBM of CuSCN was observed at 0.40 eV from the Fermi level in all samples. This shows the p-type characteristic, considering that the band gap of CuSCN is 3.65 eV [42]. The ionization energies of all CuSCN films were similar at approximately 5.4–5.5 eV. Therefore, no significant changes in the valence electronic structures were induced by antisolvent treatment, which was in good agreement with the XPS results.

**Figure 10:**
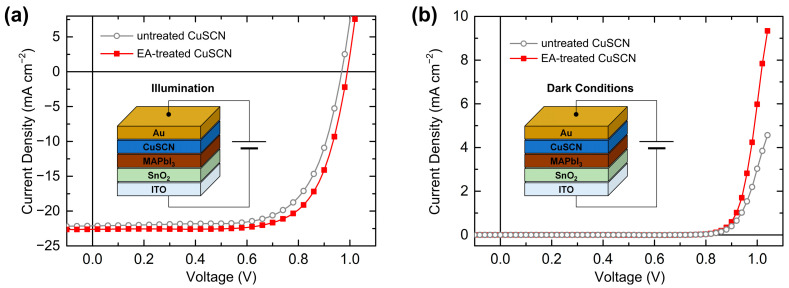
Device structure and J–V curves of the best PSCs with untreated and EA-treated CuSCN HTL in (**a**) AM 1.5G 1 sun illumination and (**b**) dark conditions.

Finally, the effect of the antisolvent treatment on the device performance was investigated by characterizing the PSCs. The SEM, XRD, XPS, and UPS results showed no significant differences among the antisolvents. Therefore, PSCs with EA-treated CuSCN HTL were representatively prepared, which has not previously been reported. The device performances of the PSCs with untreated and EA-treated CuSCN HTLs were compared. Figure 10a shows the n-i-p PSC structure of ITO/SnO_2_/MAPbI_3_/CuSCN/Au and the J–V curves of the optimal PSCs under illumination. The solar cell parameters and statistics are listed in Table 1. The PSC with untreated CuSCN HTL exhibited a short-circuit current density (J_SC_) of 22.13 mA cm^−2^, open-circuit voltage (V_OC_) of 0.97 V, and fill factor (FF) of 68.7%, resulting in a PCE of 14.72%. However, the PSC with the EA-treated CuSCN HTL showed a J_SC_ of 22.62 mA cm^−2^, V_OC_ of 0.99 V, and FF of 70.9%, yielding a PCE of 15.86%. Therefore, the EA antisolvent treatment of CuSCN mainly improved the FF, which is closely related to charge transport. This enhancement is in good agreement with previous results of improved PSCs with antisolvent-treated CuSCN HTLs [29]. Figure 10b shows the J–V curves of the same PSCs in dark conditions. At the same voltage, the J of the EA-treated CuSCN device was higher than that of the untreated CuSCN device. For example, at 1.0 V, the J of the EA-treated CuSCN device was 6.0 mA cm^−2^, while that of the untreated CuSCN device was 3.0 mA cm^−2^. Therefore, it can be concluded that the origin of improved PSC performance is improved hole transport owing to both the increased crystallinity of CuSCN and reduced damage to the perovskite layer. Given similar film properties, other antisolvent-treated CuSCN HTL PSCs are expected to show comparable performance improvements.

## 3. Materials and Methods

The ITO substrates (AMG, Uiwang, South Korea) were cleaned by ultrasonication in a sequence of deionized (DI) water, detergent, acetone, methanol, and DI water, followed by drying with nitrogen gas. The substrates were then treated with ultraviolet ozone (UV-O_3_) for 15 min at 100 °C using a PSDP-UV4T cleaner (Novascan Technology Inc., Boone, IA, USA). A 15% aqueous SnO_2_ dispersion (Alfa Aesar, Ward Hill, MA, USA) was diluted by mixing with an equal amount of DI water and stirred overnight at room temperature. The SnO_2_ was then filtered through a hydrophobic polytetrafluoroethylene membrane (pore size: 0.45 μm) and spin-coated onto the cleaned ITO substrates at 3000 rpm for 30 s, followed by annealing at 150 °C for 10 min.

The MAPbI_3_ perovskite precursor was prepared by dissolving 461 mg PbI_2_ (99.999% purity, Alfa Aesar, USA) and 159 mg methylammonium iodide (Greatcell Solar Materials Pty Ltd., Queanbeyan, Australia) in 0.6 mL N,N-dimethylformamide (99.8% purity, Sigma-Aldrich, St. Louis, MO, USA) and 0.071 mL DMSO (≥99.5% purity, Sigma-Aldrich, USA) and stirred overnight at room temperature. The perovskite solution was then spin-coated onto the ITO/SnO_2_ substrates at 4000 rpm for 30 sec. Eight seconds after reaching maximum speed, 0.6 mL of ethyl acetate (EA, 99.8% purity, Sigma-Aldrich, USA) antisolvent was dripped onto the center of the sample. Samples were heated at 150 °C for 2 min.

CuSCN (98% purity, Sigma-Aldrich, USA) was dissolved in DES (98% purity, Sigma-Aldrich, USA) at 35 mg mL^−1^ and stirred overnight at room temperature. The CuSCN solution was filtered through a PTFE membrane and spin-coated onto the ITO/SnO_2_/MAPbI_3_ at 5000 rpm for 35 sec. During spin coating, 0.1 mL of antisolvent [MA (99.5% purity, Sigma-Aldrich, USA), EA, IPA (99.5% purity, Sigma-Aldrich, USA), DE (≥99.9% purity, Sigma-Aldrich, USA), and CB (99.9% purity, Sigma-Aldrich, USA)] was dripped onto the center of the sample 15 sec after reaching maximum speed. The resulting ITO/SnO_2_/MAPbI_3_/CuSCN layers were annealed at 50 °C for 10 min. All deposition processes were performed under ambient conditions.

For PSC device fabrication, the samples were transferred to a high-vacuum chamber with a base pressure of 2 × 10^−6^ Torr, where a 90 nm Au anode (Taewon Science Co., Seoul, Republic of Korea) was deposited by thermal evaporation at a rate of 0.05 nm s^−1^. The device area was 2 × 2 mm².

SEM images were captured using an S-4800 microscope (Hitachi High-Tech Co., Tokyo, Japan), while UPS and XPS measurements were performed using a PHOIBOS 150 electron analyzer (SPECS GmbH, Berlin, Germany) with a He Iα discharge lamp (hν = 21.22 eV) and an Al Kα X-ray source (hν = 1486.7 eV). XRD patterns were obtained using a D8 Discover system (Bruker AXS GmbH, Karlsruhe, Germany). The device’s performance was measured with a Keithley 2400 source measure unit (Tektronix Inc., Beaverton, OR, USA) under AM 1.5G 1 sun illumination from a SimuLight SS-LED50S solar simulator (McScience Inc., Suwon, Republic of Korea).

## 4. Conclusions

In this study, we investigated the effect of antisolvent treatment during the spin-coating of CuSCN in n-i-p PSCs. Top-view SEM images showed that the antisolvent treatment increased the grain size of the CuSCN film. Although the cross-sectional SEM images did not show the damaged interface, the XRD results showed that the DES solvent for CuSCN degraded the underlying MAPbI_3_ perovskite layer. In addition, improved crystallinity of CuSCN was observed, which was in good agreement with the SEM results. These effects were not observed in the ITO/CuSCN system, indicating that changes in the interactions between CuSCN and MAPbI_3_ were significant. Meanwhile, the XPS spectra showed that the antisolvent treatment did not induce any additional chemical bonds or solvent residues. The UPS spectra showed no significant changes in the work function or VBM. No difference in the effect on CuSCN and MAPbI_3_ was observed between the EA, MA, IPA, DE, and CB antisolvents. As a result, the PCE of PSCs was increased from 14.72 to 15.86% via EA treatment. The increased PCE was attributed to facilitated hole transport owing to the improved crystallinity of CuSCN and MAPbI_3_. Therefore, antisolvent treatment is an efficient strategy for improving the functionality of the CuSCN HTL, which can be an alternative to the unstable and expensive spiro-OMeTAD HTL.

## Figures and Tables

**Table 1:** Solar cell parameters of PSCs with untreated and EA-treated CuSCN HTL. Data were obtained from 5 devices (average values and standard deviations shown in parentheses).

HTL	J_SC_ (mA cm^−2^)	V_OC_ (V)	FF (%)	PCE (%)
Untreated CuSCN	22.13 (22.00 ± 0.11)	0.97 (0.96 ± 0.01)	68.7 (63.90 ± 3.78)	14.72 (13.50 ± 0.92)
EA-treated CuSCN	22.62 (22.04 ± 0.49)	0.99 (0.96 ± 0.02)	70.9 (68.36 ± 1.72)	15.86 (14.45 ± 0.93)

## Data Availability

The data presented in this study are available upon request from the corresponding author.
